# Cognitive, Affective, and Feedback-Based Flexibility – Disentangling Shared and Different Aspects of Three Facets of Psychological Flexibility

**DOI:** 10.5334/joc.120

**Published:** 2020-09-09

**Authors:** Dominik Kraft, Lena Rademacher, Cindy Eckart, Christian J. Fiebach

**Affiliations:** 1 Department of Psychology, Goethe University Frankfurt, Frankfurt am Main, DE; 2 Department of Psychiatry and Psychotherapy, University of Lübeck, Lübeck, DE; 3 Brain Imaging Center, Goethe University Frankfurt, Frankfurt am Main, DE

**Keywords:** cognitive flexibility, affective flexibility, feedback-based flexibility, switching, executive function

## Abstract

Cognitive flexibility – the ability to adjust one ´s behavior to changing environmental demands – is crucial for controlled behavior. However, the term ‘cognitive flexibility’ is used heterogeneously, and associations between cognitive flexibility and other facets of flexible behavior have only rarely been studied systematically. To resolve some of these conceptual uncertainties, we directly compared cognitive flexibility (cue-instructed switching between two affectively neutral tasks), affective flexibility (switching between a neutral and an affective task using emotional stimuli), and feedback-based flexibility (non-cued, feedback-dependent switching between two neutral tasks). Three experimental paradigms were established that share as many procedural features (in terms of stimuli and/or task rules) as possible and administered in a pre-registered study plan (N = 100). Correlation analyses revealed significant associations between the efficiency of cognitive and affective task switching (response time switch costs). Feedback-based flexibility (measured as mean number of errors after rule reversals) did not correlate with task switching efficiency in the other paradigms, but selectively with the effectiveness of affective switching (error rate costs when switching from neutral to emotion task). While preregistered confirmatory factor analysis (CFA) provided no clear evidence for a shared factor underlying the efficiency of switching in all three domains of flexibility, an exploratory CFA suggested commonalities regarding switching effectiveness (accuracy-based switch costs). We propose shared mechanisms controlling the *efficiency* of cue-dependent task switching across domains, while the relationship to feedback-based flexibility may depend on mechanisms controlling switching *effectiveness*. Our results call for a more stringent conceptual differentiation between different variants of psychological flexibility.

## Introduction

Cognitive flexibility, i.e., the ability to instantaneously and flexibly adjust one’s behavior and thoughts to changing environmental demands describes a key characteristic of cognitive control and is therefore crucial for daily functioning and the pursuit of short- or long-term goals (Miyake et al., 2000; Scott, 1962). However, cognitive flexibility is often used as an umbrella term to describe different kinds of psychological constructs, leading to heterogeneous operationalizations of the concept itself (Ionescu, 2012). Empirical investigations of cognitive flexibility strongly focus on experimental paradigms involving instructed task switching. That is, participants are explicitly instructed by a cue stimulus to switch between two (or sometimes more, e.g., Piguet et al., 2013) task rules applied to the same type of stimulus. As this requires the active suppression of the ‘old’ (now irrelevant) task rule while the ‘new’ and currently relevant task rule has to be activated, task switching is cognitively more demanding then repeating the same task (Vandierendonck et al., 2010). This leads to performance costs measurable in prolonged response times and decreased performance accuracy, typically referred to as ‘switch costs’ (Jersild, 1927; Rogers & Monsell, 1995). Increased switch costs (and thus impairments in cognitive flexibility) are often reported in people suffering from psychiatric disorders (e.g., depression, Lee et al., 2012) and have been discussed as accounting for characteristic psychopathology (e.g., rumination in the case of depression; Stange et al., 2017). Between-person variability in switch costs has been reported in healthy samples as well (e.g., Armbruster et al., 2012), which we have in earlier work interpreted as reflecting individual differences in the efficiency of adjusting behavior to changing environmental demands (Ueltzhöffer et al., 2015).

As the ability to manage one’s emotions flexibly is a key characteristic of everyday life and interpersonal exchanges (e.g., Pessoa, 2008), recent work has begun to distinguish between purely *cognitive flexibility* and *affective flexibility*, i.e., the flexible engagement and disengagement from emotionally relevant information and events. Affective flexibility has been investigated with experimental paradigms involving instructed switches between affective and affectively neutral materials and/or tasks (Genet et al., 2013; Genet & Siemer, 2011; Malooly et al., 2013; Reeck & Egner, 2015).

Difficulties when detaching from negative emotional materials have in this context been associated with psychological dysfunction (e.g., Genet et al., 2013). However, prior studies directly comparing behavioral markers of cognitive and affective flexibility have so far yielded heterogeneous results, ranging from significant (Malooly et al., 2013) to no or only minor correlations (Genet et al., 2013; Genet & Siemer, 2011). Clear evidence whether these two facets of flexibility describe similar or different processes, thus, is so far lacking.

Unlike the instructed task switches discussed so far, the flexible adaptation of behavior in naturalistic situations often requires the processing of dynamically changing feedback from the environment – an ability that is disturbed in many psychiatric (e.g., Tchanturia et al., 2012) and neurological (e.g., Tisser et al., 2007) populations. These problems in flexible behavioral adaptation are also often referred to as impairments in cognitive flexibility (e.g., Lange et al., 2016), and are in clinical populations commonly assessed with tasks like the Wisconsin Card Sorting Test (WCST; Heaton, 1981). In the WCST, participants are asked to sort cards according to one of three potential rules (color, shape, number), depending on the feedback they receive from the experimenter. Flexibility is required when changing feedback indicates shifts in response-outcome mappings. In experimental studies, this type of flexible behavior is often investigated using reversal learning paradigms, which involve a discrimination phase (in which subjects learn an association between stimulus, response, and reward) and subsequent reversals of the stimulus-outcome association, to which participants have to adapt (Izquierdo et al., 2017). In the following, we distinguish this type of *feedback-based flexibility* (which in the context of clinical research is often considered “paradigmatic” for cognitive flexibility; e.g., Waltz, 2017) from externally cued task switching (i.e., cognitive flexibility) and from affective flexibility. There is an ongoing debate whether feedback-based and cognitive flexibility describe two distinct constructs or whether feedback-based flexibility constitutes a sub-category of cognitive flexibility as, e.g., proposed by Izquierdo and colleagues (2017).

There has so far been little effort to systematically investigate how potentially different constructs of behavioral, cognitive or (more generally) psychological flexibility relate to each other and whether they measure the same underlying cognitive function (Ionescu, 2012). To fill this gap, we here assessed cognitive, affective, and feedback–based flexibility in the same group of participants in a within-subjects design and investigated correlational relationships among these three facets of psychological flexibility.

Using as starting point an established paradigm for cue-based cognitive task switching (see Armbruster et al., 2012; Armbruster-Genç, Ueltzhöffer, & Fiebach, 2016; Ueltzhöffer et al., 2015), we developed two new paradigms that share as many task parameters as possible, for assessing (i) affective flexibility (using affective stimuli and switches between an emotional and a neutral judgment task) and (ii) feedback-based flexibility. To study feedback-based flexibility, we combined the two task rules from the cognitive flexibility paradigm with task reversals indicated by a change in feedback, so that participants were implicitly (rather than explicitly) instructed about task switching. We assessed the degree of shared variance among behavioral indices of cognitive, affective, and/or feedback-based flexibility. The study plan and all hypotheses were pre-registered at https://osf.io/8f4cn.

In detail, we hypothesized, first, that switch costs elicited during cognitive task switching are positively correlated with switch costs elicited in the affective flexibility task, both for response times (RT; Hypothesis 1a) and error rates (ER; Hypothesis 1b). Switch costs in the affective paradigm are calculated separately for the two tasks (i.e., for switching from the emotion to the gender task and vice versa) and hypotheses are tested for each task. In the cognitive flexibility paradigm, there is also an ambiguous condition in which the task is not explicitly cued; here, the rate of spontaneous switching serves as an additional indicator of individual differences in cognitive flexibility. Replicating Armbruster et al. (2012), we expected a negative correlation between RT switch costs and the spontaneous switch rate in the cognitive paradigm, and we furthermore hypothesized that the spontaneous switch rate (cognitive flexibility paradigm) should be negatively correlated with RT switch costs in the affective task (Hypothesis 1c).

Based on the assumption of shared underlying cognitive mechanisms, we furthermore hypothesized that the mean number of reversal errors in the feedback-based flexibility paradigm is positively correlated to RT (Hypothesis 2a) and ER (Hypothesis 2b) switch costs in the cognitive paradigm, negatively correlated with the spontaneous switch rate (Hypothesis 2c), and positively correlated with affective RT (Hypothesis 3a) and ER (Hypothesis 3b) switch costs. Following the assumption of common underlying cognitive mechanisms, we predicted that the aforementioned indices of individual differences in different facets of flexibility should load on a common underlying (i.e., latent) factor (Hypothesis 4). This will be tested using a confirmatory factor analysis. Lastly, by generalizing results from a pilot study for the affective flexibility task, we hypothesized that the RT switch costs in the cognitive paradigm will be correlated with the ER when switching to the emotion task (Hypothesis 5a), but not when switching to the gender task (Hypothesis 5b).

## Methods

### Participants

In total, N = 100 participants from a student population were included in the study (50 females, 18–35 years, mean age 23.7 ± 3.8, native speakers of German with no self-reported neurological disorders) and reimbursed with 20 Euro. 80 participants reported no history of psychiatric disorders, 10 participants reported at least one episode of psychiatric disorders in their lifetime, and 10 further participants did not provide information. Sample size was based on an a-priori power analysis for detecting small to moderate effects in a correlation analysis (*r* = .25, *p* = .05, power = .80, one tailed), using the G*Power software (Faul, Erdfelder, Buchner, & Lang, 2009). Note that this minimal effect size of interest was motivated by our hypothesis of a common underlying mechanism of psychological flexibility shared by all three paradigms. This power analysis yielded a minimum sample size of N = 97. Participants provided written informed consent and all procedures were approved by the Ethics Committee of the Department of Psychology of Goethe University Frankfurt, Germany.

### Experimental Procedures

All participants underwent the same three experimental paradigms measured within one experimental session. Prior to each paradigm, participants were trained for the respective task rules; first independently and then in task-specific combined training runs resembling the actual experimental runs. The order of paradigms was counterbalanced across individuals. For each paradigm, four pseudo-randomly ordered trial sequences were generated and equally distributed across participants. Participants were seated in a darkened room in front of a 22” monitor, and responded with their left and right middle and index fingers. Since we were interested in individual differences, the hand–task assignment for all participants was kept constant to avoid introducing additional sources of variance (Goodhew & Edwards, 2019). Responses were logged from four buttons on a regular PC keyboard; experiments were controlled using Presentation Software (Version 18.3, Neurobehavioral Systems, Inc., Berkeley, CA, www.neurobs.com).

#### Cognitive Flexibility

**Figure 1:**
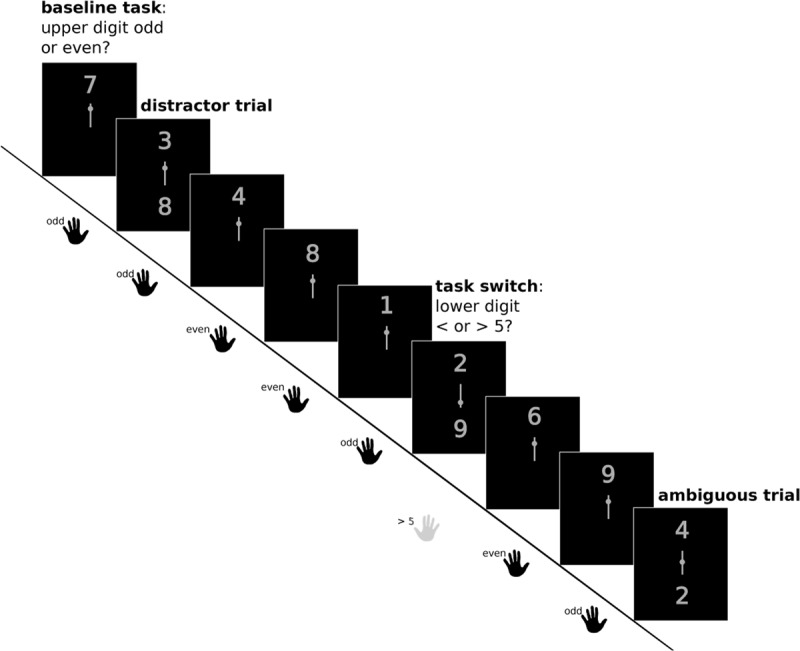
**Cognitive flexibility paradigm, adapted from (Armbruster et al., 2012).** Participants perform a baseline task (i.e., judging whether the presented digit is odd or even) and respond with their left hand. In task switch trials, participants are asked to perform a different task (i.e., judging whether the presented digit is greater or lower than 5) and now need to respond with the right hand (highlighted in grey). Whether or not participants switch task when two digits are presented depends on the placement of the small dot on the centrally presented cue bar. In ambiguous trials, participants are not unambiguously cued as to whether they should switch or not. Behavior in these trials is used to estimate the rate of spontaneous switching. After a task switch, distractor or ambiguous trial, participants continue to perform the ongoing task.

We used an adaptation of the paradigm introduced by Armbruster et al. (2012) which requires instructed switches between classifying digits (1 to 9, excluding 5) as either odd or even (parity judgment) or as higher or lower than five (magnitude judgment; see Figure 1). The two tasks are arranged in an oddball design: In 80% of trials (baseline condition), a single digit is presented in grey font against a black background above a centrally presented task cue, and participants are required to conduct an odd-even discrimination. In 20% of the trials (the critical trials), a second digit appears below the task cue. The task cue is a vertical line and the relative position of a simultaneously presented dot on that line indicates which digit participants should respond to. Critical trials are equally divided into three task conditions: If the dot is located in the upper third of the cue line, participants are trained to ignore the lower digit and to respond to the upper digit (‘distractor inhibition’ trials). In the second third of trials, the dot is located in the lower third of the cue line, indicating to switch attention to the lower digit, change task rule (i.e., </>5), and the response hand (i.e., from left to right). We refer to these trials as ‘task switching’ trials. In the last third of critical trials, the task cue is presented so close to the center of the cue line (±1 px), that it is impossible to determine whether it was located in the upper or the lower half. As participants have to reach a decision even when they are not sure about which digit to respond to, this ambiguous condition can reflect their tendency towards more or less flexible behavior. After every critical trial, participants continue to perform the baseline task for three to six trials. For further information about the cognitive flexibility paradigm, see Figure 1 and Armbruster et al. (2012).

The cognitive flexibility experiment was 10 minutes and 6 seconds long and comprised two runs of 150 trials each, so that in total each subject completed 240 baseline trials and 20 trials in each of the three critical task conditions. Each trial lasted for 2,000 ms. Stimulus presentation started with trial onset and lasted for 1,000 ms, while responses were registered during the whole trial period. Switch costs were calculated by subtracting the subject-specific mean RT or ER during baseline trials from the mean during switch trials.

The spontaneous switch rate was computed as the number of hand switches (which are indicative of an intended task switch) in ambiguous trials, divided by the number of ambiguous trials.

#### Affective Flexibility

To investigate switching to and from affective materials, we developed a new paradigm that is a combination of the above-described cognitive flexibility task and an affective task switching paradigm described by Dierolf and colleagues (2016). The stimulus set comprised pictures of 120 persons (60 female, 60 male) drawn from the FACES database (Ebner et al., 2010), each included twice (i.e., with a happy and an angry facial expression), resulting in a total of 240 stimuli. We chose those 60 male and female pictures from the database for which Ebner et al. (2010) report the best accuracy ratings for the emotion ‘angry’ (ranging from 83% to 100%; overall mean 90,92%).

**Figure 2:**
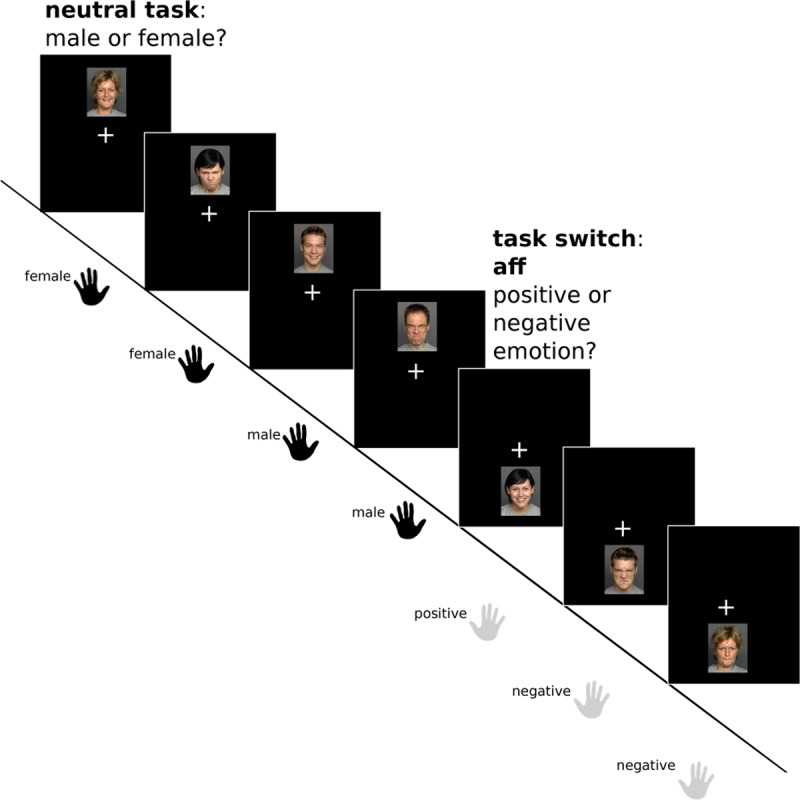
**Affective flexibility paradigm.** Participants perform either an affectively neutral task (i.e., judging whether the presented face is male or female; response with left hand) or a task focusing on the affective content of the stimulus images (i.e., judging whether the face shows a positive or negative emotion; response with right hand). The to-be-performed task depends on the location of the stimulus (above vs. below the fixation cross, respectively). After a switch, participants continue to perform the new task (repetition trials) until the next switch trial occurs (highlighted in grey).

Participants had to switch between a gender judgment (female vs. male; neutral task) and a valence judgment (positive vs. negative emotional expression). The position of the image on the screen served as implicit task cue – stimuli above the fixation-cross indicated the gender task, stimuli below the fixation-cross indicated the emotion task. This position–task assignment was held constant across all trials. Participants worked on the respective task for 3 to 7 trials, until a change in the position of the image indicted a task switch, after which participants continued to perform the ‘new’ task until the next switch was cued by another positional change. Unlike in the cognitive flexibility task, no distractor or ambiguous conditions were included in this paradigm, in order to reduce complexity and length of the experiment. The affective flexibility paradigm consisted of one run of 240 trials – 24 switch trials and 96 baseline (repetition) trials for each task condition – and had a total length of 8 minutes and 3 seconds. Consistent with the cognitive flexibility task, each trial lasted for 2,000 ms and stimuli were presented for 1,000 ms with responses being logged during the whole trial period (see Figure 2). RT and ER switch costs were calculated in analogy to the cognitive flexibility task (switch minus repeat trials). However, here we distinguished between switches from the neutral gender to the emotion task and vice versa, to identify potential differences in switching towards vs. away from affective information. Importantly, we used repetition trials of the same task to calculate the respective switch costs (e.g., RT switch costs to gender were calculated by subtracting mean RT in gender repeat trials from mean RT in switch trials to the gender task).

#### Feedback-Based Flexibility

**Figure 3:**
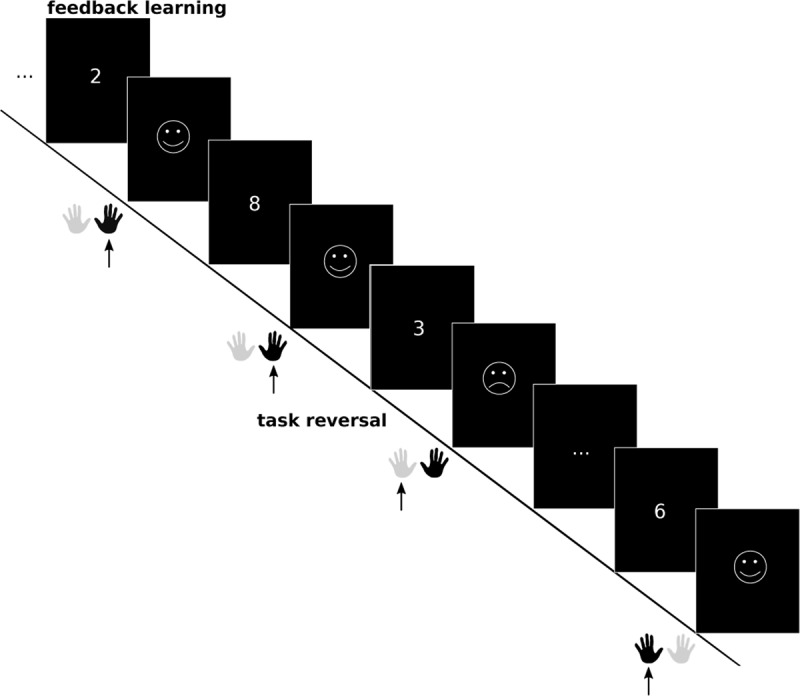
**Feedback-based flexibility paradigm.** Participants either perform the odd/even judgment task or the </>5 task (see Figure 1). Participants need to learn the current rule based on the visual feedback they receive after each trial, and have to adjust their behavior after rule reversals. Probabilistic errors are not shown in the schematic, but involve a negative feedback without an actual task reversal, and serve to increase the unpredictability of task reversals. The dark hand indicates the participant’s button press, while the arrow points toward the correct hand (i.e., rule) to use.

To quantify the ability to dynamically adjust behavior in response to changing feedback, a novel paradigm was developed by combining feedback-based reversals (e.g., Cools et al., 2002) with the same task rules used in the cognitive flexibility paradigm (i.e., odd/even and </>5, mapped to different hands). Participants were not explicitly cued about a required task-switch but had to learn the current rule based on feedback. Single digits (1 to 9, except 5) were presented in the middle of the screen (white on black background), and participants received positive or negative feedback (laughing or sad smiley, respectively) depending on their response. After 6–15 trials of the same task, an uninstructed task reversal occurred, i.e., the underlying rule changed. Now, responses according to the former task rule resulted in negative feedback (see Figure 3). The mean number of errors after a task reversal (i.e., the number of errors until responses are in accordance with the new rule) served as indicator for individual differences in feedback-based flexibility – fewer errors after rule reversals are indicative of greater flexibility.

To increase cognitive demand and uncertainty about the time of the reversal, and to prevent participants from using simple win-stay/loose-switch strategies, we included probabilistic errors in 20% of the sequences of trials between two task reversals. In these cases, a correct response was followed by negative feedback, even though no task reversal had occurred. Probabilistic errors occurred between actual reversals, but never in the first four trials after a reversal or in the last trial before a reversal. As this limits the occurrence of the probabilistic error to a single position for the shortest inter-reversal intervals (with a length of six trials), these sequences never contained a probabilistic error to avoid participants noticing any regularity. The feedback-based flexibility paradigm comprised one experimental run of 109 trials with 10 reversals, resulting in a total duration of 7 minutes and 33 seconds. Stimuli were presented for 1,500 ms each and feedback was given for 500 ms, resulting in a total trial length of 2,000 ms. Trials were followed by a jittered inter-trial interval (ITI; 1,000–6,000 ms), and a fixation cross was presented in the middle of the screen for the last 500 ms of the ITI, to prepare for the upcoming trial.

### Exclusion criteria

We had preregistered that participants with error rates higher than 30% in at least one condition of the cognitive or affective flexibility paradigms would be excluded from all analyses relating to the respective paradigm. However, for the cognitive paradigm with its three relevant conditions (i.e., baseline, switch, and ambiguous trials), this would have led to the exclusion of 20 participants, i.e., one fifth of the sample. Therefore, we decided to exclude participants condition-wise, such that participants with error rates >30% in one condition were only excluded from analyses involving that condition. For analyses of the feedback-based flexibility paradigm, subjects were excluded if they never had switched rules. Due to the aforementioned exclusion procedure, sample sizes differed between analyses and ranged from 82 to 99 subjects (see results in Tables 3 and 4). Following our preregistration, RTs below 250ms and greater than three standard deviations (SD) above the person’s mean of the respective condition were considered outliers and were therefore excluded. Error trials were excluded from RT analyses in the cognitive and affective flexibility paradigm.

### Statistical Analyses

Statistical analyses were performed as pre-registered; deviations from the pre-registered protocol are explicitly indicated.

#### Task Parameters and Flexibility Indices

For cognitive and affective flexibility, we calculated repeated measures ANOVAs to investigate the effect of task condition (cognitive: baseline vs. switch vs. ambiguous trials; affective: emotion vs. gender task × repetition vs. switch trials) for both RT and ER (Pingouin package, Version 0.2.1, Vallat, 2018). Where appropriate, significant effects were further resolved using paired post-hoc t-tests (Bonferroni corrected for multiple comparisons; Bonferroni, 1936). In the affective task, post-hoc tests served to examine whether switch costs differ between shifts towards emotion vs. gender task. For the feedback-based flexibility paradigm with only one main parameter of interest (the number of consecutive errors after a task reversal), we did not calculate any inferential statistics. Furthermore, mean values, SD, and 95% confidence intervals (CI) were calculated for the flexibility indices for each paradigm.

#### Psychometrics of Flexibility Indices

Internal consistencies for cognitive and affective flexibility were calculated via permutation-based split-half reliability estimates, which were obtained separately for RT and ER switch cost measures using the splithalf package (version 0.7.1; Parsons, 2020) for R (version 3.5.2; R Core Team, 2018); as described above, sample sizes could differ slightly between tasks. Obtaining an estimate of internal consistency for the spontaneous switch rate in the ambiguous trials of the cognitive paradigm is not straightforward. At the single-trial level, participants’ responses in ambiguous trials can be represented as 0 (i.e., stay) or 1 (i.e., switch). However, applying random splits in a permutation-based framework on this kind of data is problematic, which we would like to illustrate with a toy example: Imagine a participant completed 20 ambiguous trials with a switch/stay proportion of 50:50, i.e., he or she switched 10 times. After randomly splitting the data, split1 contains 6 and split2 4 switch trials. The allocation of switch trials into split1 and split 2 is therefore ‘deterministic’, as the number of switch trials in split 1 determines how many switch trials can be allocated to split2 and vice versa. Consequently, any summary score calculated for split1 and split2 will be (almost) complementary. With a sufficient amount of iterations, the estimate of internal consistency (i.e., the correlation between split 1 and split 2), converges to 1 – not describing a perfect reliability, but rather data idiosyncrasy. To overcome this issue, we decided against randomly splitting the data and calculated the correlation between the number of switches for the first half of the trials and the second half of the trials per subject. This might serve as a proxy and an easy and interpretable measure of subjects’ response contingency (MacDonald & Trafimow, 2013) for which we do not report Spearman-Brown corrected values given its potential imprecision.

Due to the nature of the flexibility index derived from the feedback-based task (i.e., the outcome measure being a sequence of errors, as a direct consequence of an implicit task reversal), internal consistency analyses were not feasible using the splithalf approach. Instead, we derived an estimate in the following way: For each participant, we obtained a list containing 10 values representing the total number of errors after each of the 10 task reversals (e.g., if a participant made three errors after the [i]^th^ task reversal, the [i]^th^ position in the list would be filled with a ‘3’), and these lists were appended to an omnibus list, allowing to iterate over participants. In each of 5,000 iterations, each of these subject-specific lists was randomly shuffled, split into two parts, means were calculated for both sub-lists, and correlations were calculated between split1 and split2. For all different approaches to obtaining split-half correlation, the final results represent averages of 5,000 random splits, which are reported as uncorrected and Spearman-Brown corrected reliability estimates, together with their respective 95% CIs.

#### Correlation Analyses

One-sided correlation analyses were calculated between flexibility measures for a-priori planned tests of directional hypotheses (H1–H3, H5) using the SciPy Statistics (stats) module (Jones et al., 2014) in Python (version 3.6.9, www.python.org). Flexibility indices were tested for normality using the Shapiro-Wilk test (Shapiro & Wilk, 1965). In case of non-normality, Spearman correlations (*r_s_*) were calculated instead of Pearson correlations (*r*). Note that this deviates from the pre-registered analysis plan, as only Pearson correlations were pre-specified. Correlations were corrected for multiple comparisons using the Bonferroni approach and are reported with a 95% CI.

#### Factor Analyses

To further investigate whether the above-described flexibility indices can be combined to one latent variable (which would be indicative of a common underlying cognitive mechanism that controls flexible behavior in general; H4), a confirmatory factor analysis (CFA) was conducted using the lavaan package (Version 0.6.3) in R (Rosseel, 2012). Bartlett test and the Kaiser-Meyer-Olkin measure for sampling adequacy were calculated to test factorization assumptions. Multivariate normality was tested using Mardia’s test (Mardia, 1980). We fitted a model with z-standardized flexibility indices (i.e., RT switch costs in the cognitive and affective paradigm, spontaneous switch rate, and mean number of reversal errors) loading onto one single factor using a robust Maximum likelihood estimator (MLM), without applying rotation.

## Results

Our main research question was to explore whether there are joint variance components between behavioral markers of flexibility from the various domains i.e., purely cognitive vs. affective vs. feedback-based flexibility. Before that, we will however describe the main parameters of the tasks (see also Table 1).

**Table 1:** Descriptive statistics for the main parameters of the cognitive and affective flexibility paradigm (N = 100).

	*M (SD)*	

	RT	ER

***Cognitive flexibility***		
baseline trials	687.96 (87.93)	.04 (.03)
task switch trials	1023.56 (167.04)	.11 (.12)
ambiguous trials	1143.30 (215.17)	.20 (.15)
***Affective flexibility***		
emotion baseline trials	708.97 (89.31)	.08 (.05)
emotion switch trials	977.78 (160.69)	.16 (.15)
gender baseline trials	652.77 (91.93)	.06 (.06)
gender switch trials	900.00 (140.84)	.07 (.10)
(overall) baseline trials	686.83 (89.38)	.07 (.05)
(overall) switch trials	935.91 (144.15)	.12 (.11)
(overall) emotion trials	758.07 (94.76)	.12 (.09)
(overall) gender trials	701.40 (95.28)	.07 (.07)

*M* = mean; *SD* = standard deviation; RT = response time in millisecond; ER = error rate.

### Task parameters and flexibility indices

For the cognitive flexibility paradigm, we observed a significant effect of task condition on RTs, *F*(2,160) = 391.11, *p* < .001, \eta _P^2 = .83, and and on ERs, *F*(2,198) = 71.74, *p* < .001, \eta _P^2 = 42. Post hoc t-tests (Bonferroni-adjusted α = .05/3 = .017) revealed significantly higher RTs in switch compared to ongoing baseline trials, *t*(80) = 23.64, *p* < .001, Hedge’s g = 2.73 (see also Table 2 for switch cost estimates). Furthermore, RTs were significantly higher in ambiguous trials – both compared to baseline trials, *t*(80) = 22.15, *p* < .001, Hedge’s g = 3.02 and switch trials, *t*(80) = 7.95, *p* < .001, Hedge’s g = .65. A similar pattern was observed for performance accuracy, with significantly higher ERs for switch, *t*(99) = 5.97, *p* < .001, Hedge’s g = .83, and ambiguous, *t*(99) = 10.98, *p* < .001, Hedge’s g = 1.70, compared to baseline trials. Furthermore, participants had higher ERs in ambiguous trials compared to switch trials, *t*(99) = 6.55, *p* < .001, Hedge’s g = .70.

**Table 2:** Descriptive statistics for flexibility indices, sorted by paradigm (N = 100).

	range	*M* (*SD*)	CI

***Cognitive flexibility***			
Switch cost RT	[76.58–724.26]	335.60 (132.39)	[309.65–361.55]
Switch cost ER	[–.10–.45]	.06 (.10)	[.04–.08]
Spontaneous switch rate	[.0–1.0]	.47 (.32)	[.40–.53]
***Affective flexibility***			
Switch cost RT (to emotion)	[84.00–624.77]	268.82 (119.26)	[245.44–292.19]
Switch cost RT (to gender)	[51.19–534.61]	247.23 (96.75)	[228.27–266.20]
Switch cost ER (to emotion)	[–.10–.54]	.08 (.12)	[.06–.11]
Switch cost ER (to gender)	[–.12–.41]	.01 (.07)	[.00–.03]
***Feedback-based flexibility***			
(mean) reversal errors	[1.00–3.20]	1.72 (.42)	[1.63–1.80]

*M* = mean; *SD* = standard deviation; RT = response time in millisecond; ER = error rate; CI = 95% confidence interval.

In the affective flexibility paradigm, participants responded significantly slower, *F*(1,99) = 156.45, *p* < .001, \eta _P^2 = .61, and less accurate, *F*(1,99) = 38.45, *p* < .001, \eta _P^2 = .28, in the emotion task compared to the gender task (Table 1). Irrespective of task (i.e., emotion vs. gender), RTs in repetition trials were significantly faster compared to switch trials, *F*(1,99) = 671,51, *p* < .001, \eta _P^2 = .87, and error rates were higher for switch compared to repetition trials, *F*(1,99) = 42.26, *p* < .001, \eta _P^2 = .30. In addition, the task x condition interaction was significant both in RTs, *F*(1,99) = 6.21, *p* = .01, \eta _P^2 = .06, and ERs, *F*(1,99) = 29.59, *p* < .001, \eta _P^2 = .23. When resolving the interaction effects (Bonferroni-adjusted α = .05/2 = .025) according to task, we observed significantly higher RT in switch trials compared to baseline trials in both tasks, i.e., for the gender task *t*(99) = 25.55, *p* < .001, Hedge’s g = 3.60, and for the emotion task *t*(99) = 22.54, *p* < .001, Hedge’s g = 3.20. For ER, we observed significantly higher ER in switch trials compared to baseline trials in the emotion task *t*(99) = 6.99, *p* < .001, Hedge’s g = .99, but not for the gender task (*p* > .05).

In the feedback-based flexibility paradigm, participants made, on average, between 1 and 3.2 errors before adopting the new task rule (mean: 1.72 errors; Table 2). As Table 2 also demonstrates, participants exhibited substantial variations in flexibility indices from all three paradigms – justifying individual differences analyses.

### Psychometrics of Flexibility Indices

We obtained (Spearman-Brown corrected) reliability estimates between *r*_SB_ = .82 and .93 for RT switch costs and between *r*_SB_ = .37 and .62 for ER switch costs. More specifically, for the cognitive flexibility paradigm, we obtained corrected reliability estimates of *r*_SB_ = .93, CI [.91, .95] for RT switch costs and of *r*_SB_ = .37, CI [.12, .56] for ER switch costs. Spearman-Brown corrected reliability estimates for the affective flexibility paradigm were (i) for RT switch cost (when switching to the emotion task) *r*_SB_ = .85, CI [.80, .89], (ii) for RT switch cost (to gender) *r*_SB_ = .82, CI [.75, .88], (iii) for ER switch cost (to emotion) *r*_SB_ = .62, CI [.49, .73], and (iv) for ER switch cost (to gender) *r*_SB_ = .43, CI [.21, .60]. For the spontaneous switch rate in the cognitive paradigm we obtained an estimate of *r* = .83, CI [.75, .89]. For the mean reversal errors in the feedback-based flexibility paradigm, we obtained a corrected reliability estimate of *r*_SB_ = .71, CI [.60– .80]. See Table 3 for further information and uncorrected estimates.

**Table 3:** Internal Consistencies for flexibility indices obtained from each paradigm.

	N	split-half	CI	*r*_SB_	CI

***cognitive***					
Switch cost RT	94	.87	[.83, .91]	.93	[.91, .95]
Switch cost ER	94	.23	[.06, .39]	.37	[.12, .56]
spontaneous switch rate	83	.83	[.75, .89]		
***affective***					
Switch cost RT (to emotion task)	99	.74	[.66, .81]	.85	[.80, .89]
Switch cost RT (to gender task)	99	.70	[.60, .78]	.82	[.75, .88]
Switch cost ER (to emotion task)	99	.45	[.32, .58]	.62	[.49, .73]
Switch cost ER (to gender task)	99	.28	[.11, .43]	.43	[.21, .60]
***feedback***					
reversal errors (*M*)	100	.55	[.40, .68]	.71	[.60, .80]

*M* = mean, N = sample size, split-half = uncorrected estimates, CI = 95% confidence interval, *r*_SB_ = Spearman-Brown corrected estimates.

### Correlation Tests

The presence of hypothesized associations between behavioral indicators of cognitive, affective, and feedback-based flexibility (H1, H2, H3), as well as potentially task-specific associations between ER switch costs in the affective flexibility task and RT switch costs in the cognitive paradigm (H5) were evaluated using one-sided correlation tests (corrected for multiple comparisons according to Bonferroni’s procedure). Point estimates and 95% confidence intervals are reported in Tables 4 and 5. As described in the Methods section, the number of observations differs between analyses depending on the exclusion criteria. However, all results also hold true when repeating the analyses with the full sample of N = 100 (Supplementary File 1, Table S1, S2).

**Table 4:** Pre-registered correlations for response time flexibility indices, spontaneous switch rate, and mean number of reversal errors.

	*affective*		*feedback*

	Switch cost RT (to emotion task)	Switch cost RT (to gender task)	reversal errors (*M*)

***cognitive***			
Switch cost RT	*r_s_* = .35	*r* = .49	*r_s_* = .17
	*p* < .001^a^	*p* < .001^a^	*p* = .05^b^
	CI = [.16–.52]	CI = [.32–.63]	
spontaneous switch rate	*r_s_* = .03	*r_s_* = .06	*r_s_* = –.12
	*p* = .39^d^	*p* = .30^d^	*p* = .13^e^
***affective***			
Switch cost RT (to emotion task)			*r_s_* = –.03
			*p* = .39^c^
Switch cost RT (to gender task)			*r_s_* = .02
			*p* = .41^c^

Due to exclusion criteria, sample size varies across correlations; all correlations are one-sided; ^a^ N = 93; ^b^ N = 94; ^c^ N = 99; ^d^ N = 82; ^e^ N = 83; RT = reaction time in millisecond; *M* = mean; CI = 95% confidence interval.

**Table 5:** Pre-registered correlations for accuracy-based flexibility indices and the mean number of reversal errors.

	*affective*		*feedback*

	Switch cost ER (to emotion task)	Switch cost ER (to gender task)	reversal errors (*M*)

***cognitive***			
Switch cost ER	*r_s_* =.17	*r_s_* = .12	*r_s_* = .01
	*p* = .05^a^	*p* = .12^a^	*p* = .45^b^
***affective***			
Switch cost ER (to emotion task)			*r_s_* = .26
			*p* < .01^c^
			CI = [.07–.44]
Switch cost ER (to gender task)			*r_s_* = –.04
			*p* = .33^c^

Due to exclusion criteria, sample size varies across correlations; all correlations are one-sided; ^a^ N = 93; ^b^ N = 94; ^c^ N = 99; *M* = mean; ER = error rate; CI = 95% confidence interval.

#### Association between cognitive and affective flexibility

**Figure 4:**
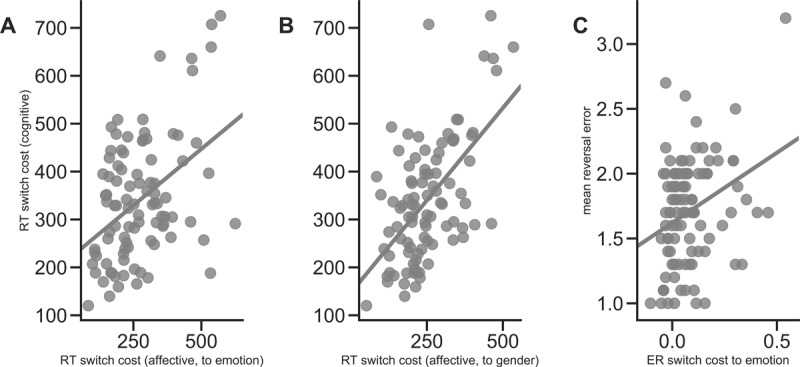
**Correlation results for preregistered hypotheses tests. (A, B)** Correlations between response time switch costs for the cognitive (y-axis) and affective (x-axis) flexibility paradigm, separated according to the direction of task switch in the affective flexibility paradigm, i.e., (A) for switches from the gender task to the emotion task and (B) for switches from the emotion to the gender task. **(C)** Correlation between mean number of errors after reversal in the feedback-based flexibility paradigm (y-axis) and error rate switch costs in the affective flexibility paradigm (while switching from the neutral task to the emotion task). RT = response times in millisecond, ER = error rate.

We found significant associations between RT switch costs in the cognitive and affective flexibility paradigms (H1a). This held true for switches from the gender task to the emotion task (*r_s_*(91) = .35; *p* < .001; Bonferroni-adjusted α = .05/3 = .017; CI = [.16–.52]; Figure 4A) as well as for switches from emotion to gender (*r*(91) = .49; *p* < .001; CI = [.32–.63]; Figure 4B). A two-tailed statistical comparison of the strengths of these two correlation coefficients (cocor; Diedenhofen & Musch, 2015) showed a tendency towards differential correlation effects, which however did not become significant, *t*(90) = 1.85, *p* = .07. Furthermore, ER switch costs in the cognitive paradigm showed a tendency towards a significant correlation with ER switch costs in the affective paradigm (H1b), at least while switching to the emotion task (*r_s_*(91) = .17; *p* = .05). However, this association did not survive correction for multiple comparisons. There was no significant association between cognitive ER switch costs and ER switch costs while participants had to switch from emotion to the gender task (Table 5). Finally, even though we replicate the correlation between spontaneous switch rate and cognitive RT switch costs reported by Armbruster et al. (2012), *r_s_*(78) = –.25, *p* = .026, this association did not generalize to RT switch costs in the affective flexibility task (H1c; Table 4). These results did not change when using partial correlations to take into account that ten participants reported occurrence of one or more psychiatric episodes in their lifetime (see Supplementary File 1, Table S3, S4).

#### Associations between feedback-based flexibility and cognitive/affective flexibility

The number of consecutive errors after a reversal in the feedback-based flexibility paradigm was correlated with affective ER switch costs when switching to the emotion task (H3b; *r_s_*(97) = .26; *p* = .004; Bonferroni-adjusted α = .05/2 = .025; CI = [.07–.44]; Figure 4C). Note that this correlation also held true when excluding the potential outlier in the upper right part of the scatter plot (*r_s_*(96) = .24; *p* = .009). However, the number of errors after reversal was not related to RT switch costs in the affective flexibility paradigm (H3a) or to measures of cognitive flexibility (H2a–c; see Tables 4 and 5). These results did not change when using partial correlations to take into account that ten participants reported occurrence of one or more psychiatric episodes in their lifetime (see Supplementary File 1, Table S3, S4).

#### Differential correlation between ER switch costs in the affective flexibility task and RT switch costs in the cognitive flexibility task

Against our expectations, affective ER switch costs did not differentially correlate with cognitive RT switch costs – neither while switching to emotion judgements (H5a) nor while switching to gender judgments (H5b).

#### Further exploratory analyses

To exclude the possibility that observed associations between RT switch costs in the affective and cognitive domain might be a side effect of general processing speed (irrespective of task switching abilities), we assessed whether RT switch costs were correlated with RTs in the respective baseline or repeat conditions. RTs in the baseline/repeat conditions, thus, in this analysis served as proxy for general processing speed, which appears valid given that they significantly correlated between the cognitive and affective paradigms (emotion task: *r_s_*(97) = .63; gender task: *r_s_*(97) = .69; both *p* < .001). However, in both paradigms, RT switch costs did not correlate with the respective baseline conditions (all *p* > .05).

To furthermore investigate the association of processing speed (RT) and accuracy, we correlated these measures for different trial types in the cognitive and affective flexibility paradigms. In the cognitive paradigm, we did not observe a speed accuracy tradeoff, *r_s_*(92) = .14, *p* > .05 in baseline trials. However RT and ER in switch trials, *r_s_*(92) = –.34, *p* < .001, as well as RT and ER switch costs, *r_s_*(92) = –.35, *p* < .001, were negatively correlated, indicating lower ER with increased RT. For the affective flexibility task, we did not find any associations between RT and ER in baseline or switch trials (all *p* > .05). However, RT and ER switch costs when switching to the gender task were positively correlated, *r_s_*(97) = .21, *p* < .05, while there was no significant association when switching in the opposite direction (*p* > .05).

Our hypothesis 5 concerning ‘cross-paradigm’ associations between cognitive RT switch costs and affective ER switch costs (motivated by results from a pilot experiment) could not be supported (see above). However, a significant negative correlation was found post-hoc between cognitive ER switch costs and RT switch costs in the affective flexibility paradigm while switching to the gender task, *r_s_*(91) = –.31, *p* = .001; Bonferroni-adjusted α = .05/2 = .025, but not while switching to the emotion task, *r_s_*(91) = –.18, *p* = .05.

### Confirmatory Factor Analysis

Confirmatory factor analysis was used to test more directly the hypothesis that shared variance between behavioral indices of cognitive, affective, and feedback-based flexibility might reflect a common underlying factor determining individual differences in flexible behavior and cognition (H4). Testing the factorization assumptions (see Table 6), Kaiser-Meyer-Olkin test revealed little shared variance within the inter-correlation matrix (Kaiser & Rice, 1974), whereas Bartlett’s test implied a significant deviation from the identity-matrix. Given the non-normally distributed data the confirmatory factor analysis was performed with a robust estimator (i.e., MLM; Curran et al., 1996). Factor loadings for the flexibility indices mirrored heterogeneous model fit findings, with all three RT-based switch cost indices loading moderately (cognitive RT switch costs = .58) to high (affective RT switch cost to emotion = .72; to gender task = .97) on the single factor, while spontaneous switch rate and reversal errors did not load on the factor (see Table 6 for further information on loadings and fit indices).

**Table 6:** Factor Loadings for single factor solution (confirmatory factor analysis).

Indicator	Beta	*SE*	*Z*	*p*

Switch cost RT (to emotion)	.72	.11	6.63	.000
Switch cost RT (to gender)	.97	.12	8.10	.000
Switch cost RT	.58	.13	4.41	.000
Spontaneous switch rate	.06	.11	.54	.59
Reversal error (*M*)	.08	.12	.65	.51

All indicators were z-standardized; Beta = standardized factor loading, SE = standard error. M = mean. Kaiser-Meyer-Olkin overall sampling adequacy = .60 [.27–.67], Bartlett test, chi^2^ = 111.99, *p* < .001, Mardia’s test (skewness, *p* < .001 & kurtosis, *p* < .01). Fit indices: comparative fit index = .94, Tucker-Lewis-Index = .89, Root-Mean-Square-Error-of-Approximation = .10, 90% CI = [.00–.19].

While Hypothesis 4 focused primarily on the efficiency of task switching in terms of RT switch costs (plus the spontaneous switch rate and reversal errors), we repeated the factor analysis also for ER switch costs in an exploratory manner. Given comparable results regarding model assumptions (i.e., factorization and multivariate normality; Table 7), factor analysis was performed as described above, resulting in descriptively better fit indices. The model yielded low to high loadings for cognitive switch costs (.28), reversal errors (.39), costs of switching to emotion (.83), while ER switch costs for switching to emotion and the spontaneous switch rate did not load on the factor. Thus, here, reversal errors and cognitive and affective switch costs did indeed load on a single factor (with the caveat that only the more difficult affective switch cost index was involved, see Table 7 for further information on loadings and fit indices).

**Table 7:** Factor Loadings for single factor solution (confirmatory factor analysis), exploratory post-hoc analysis.

Indicator	Beta	*SE*	*Z*	*p*

Switch cost ER (to emotion)	.83	.24	3.50	.000
Switch cost ER (to gender)	.18	.16	1.08	.279
Switch cost ER	.28	.13	2.26	.02
Spontaneous switch rate	–.03	.13	–.23	.82
Reversal error (*M*)	.39	.18	2.21	.03

All indicators were z-standardized; Beta = standardized factor loading, SE = standard error. M = mean. Kaiser-Meyer-Olkin overall sampling adequacy = .56 [.49–.64], Bartlett test, chi^2^ = 21.20, *p* = .02, Mardia’s test (skewness & kurtosis, both *p* < .001). Fit indices: comparative fit index = 1.0, Tucker-Lewis-Index = 1.73, Root-Mean-Square-Error-of-Approximation = .00, 90% CI = [.00–.10].

## Discussion

To resolve conceptual heterogeneity associated with the term cognitive flexibility (e.g., Ionescu, 2012) and to systematically explore associations with affective flexibility, the present study investigated inter-relations among three different facets of flexible thought and behavior, i.e., cognitive, affective, and feedback-based flexibility. Results partially support our first hypothesis, i.e., that the efficiency of switching in a purely cognitive vs. in an affective task domain are related. This result was restricted to response time switch costs (Hypothesis 1a) and did not generalize to error rate costs (Hypothesis 1b) or the rate of spontaneous switching in the cognitive paradigm (Hypothesis 1c).

Furthermore, we received partial support for our hypothesis that feedback-based flexibility (realized as the feedback-dependent switching between two task sets, in a paradigm closely modeled after probabilistic reversal learning tasks; e.g., Cools et al., 2002) is also related to the other two domains of flexibility examined here: Inflexibility in feedback-dependent task switching (i.e., higher numbers of reversal errors before switching) correlated with higher ER switch costs in the affective paradigm (Hypothesis 3b) – at least when switching from the affectively neutral gender task to the emotion task. Moreover, a tendency towards a similar correlation emerged between the number of reversal errors and RT switch costs in the cognitive task (Hypothesis 2a), even though it did not reach statistical significance. The confirmatory factor analysis did not support our strong hypothesis of a clearly identifiable common mechanism underlying the efficiency of flexible behavior and cognition (in terms of RT switch costs; Hypothesis 4), but an exploratory analysis shows significant loadings of cognitive and affective ER switch costs as well as the number of reversal errors on a shared latent factor, possibly suggesting a shared mechanism controlling the effectiveness (as opposed to efficiency) of task switching across domains.

### Associations between cognitive and affective task switching

Our observation of shared mechanisms between cognitive and affective flexibility is partly consistent with results from a previous series of studies that, however, did not focus explicitly on the association between cognitive and affective flexibility: Malooly and colleagues (2013) report a significant correlation between cognitive and affective switch costs while Genet et al. (2013) more selectively observed a positive correlation between cognitive RT switch costs and switching from the affective to the neutral task rule given a positive image. A third study from the same authors found no association between cognitive and affective RT switch costs (Genet & Siemer, 2011). This may reflect a lack of statistical power as this study had the smallest sample size (but the correlation pointed descriptively in the same direction as our result). Malooly et al. (2013) and Genet et al. (2013) used comparably large samples and report correlations separately for four different types of switches (i.e., to the affective vs. to the neutral task and to positive vs. negative images). In doing so, they obtained different correlation results across the two studies, which may reflect that trial numbers in the specific conditions became too low to obtain replicable results. However, wherever these authors observed significant correlations, they were in the same direction as in the present study. Despite these partly heterogeneous results from previous studies, we here stress the robustness of our present correlation results in a sufficiently powered sample, and thus interpret our findings as converging evidence for the presence of an association between cognitive and affective switch costs. Consistent with the observation of ‘asymmetric switch costs’ between more and less dominant task sets (Monsell et al., 2000), switching was easier when switching to the (emotionally less salient and dominant) gender task (see Reeck & Egner, 2015, for converging evidence and discussion). However, despite this asymmetric affective switch cost effect, the difference in effect sizes of the correlations between cognitive and affective switch costs depending on the direction of the affective switch (i.e., to emotional vs. to neutral) did not reach significance. We interpret this as suggesting a shared, rule-independent mechanism involved in both cognitive and affective flexibility. A potential caveat to this conclusion would be that the correlation between switch costs might reflect superficial task similarities, in particular the fact that speeded response times in both tasks might depend on between-person differences in basic processing speed. However, the fact that switch costs did not correlate with respective response times in baseline/repetition trials (while baseline RTs did correlate between paradigms) does not seem to be compatible with this alternative interpretation. Furthermore, there was no evidence of a general speed-accuracy trade-off in baseline trials, neither in the cognitive nor in the affective flexibility task.

The weaker or absent correlations between cognitive and affective ER switch costs may very well be a result of the low to moderate reliabilities we obtained for these ER-based flexibility measures, as reliability influences the observable correlation between to variables (Hedge et al., 2018). It has been argued that error rates generally lack the condition-related specificity necessary for creating reliable difference scores (Miller & Ulrich, 2013), as error probabilities might be determined more by common factors (like attention or motivation) that affect both experimental conditions (repeat and switch), instead of reflecting task-specific mental processes. Moreover, the very small accuracy switch cost effect when switching from the affective task to the gender task (i.e., 1%) additionally indicates a lack of sufficient between-subject variance in the data for such associations to emerge. In contrast, reliability estimates for RT switch costs were very good to excellent (e.g., Koo & Li, 2016), lending credibility to the analysis of individual differences and correlations in RT-based flexibility measures.

### Feedback-based vs. cue-instructed flexibility

Neuropsychological tests requiring feedback-dependent changes of the task set, like the Wisconsin Card Sorting Test, are often also interpreted as assessing deficits in cognitive flexibility (e.g., Kalia et al., 2018; Waltz, 2017). To more systematically clarify the relationships between feedback-dependent and cue-instructed cognitive flexibility (see also the more detailed and broader discussion in Ionescu, 2012), and to explore possible associations with affective flexibility, participants also completed a task that required task switching according to feedback about their task performance (rather than explicit pre-stimulus cueing of the task). Results concerning the association between instructed and feedback-based task switching were less clear than those for the association between cognitive and affective flexibility: The number of errors after reversal, i.e., our measure of individual differences in feedback-based flexibility, correlated only marginally with cognitive RT switch costs, and it did not load on the latent factor that showed associations with cognitive and affective RT switch costs (in the preregistered confirmatory analysis). Even though descriptively, subjects with longer RT switch costs also made more errors before behavioral adaption to the new rule, this result cannot be considered strong evidence in favor of an association. On the other hand, there was a significant correlation between the number of errors after reversal and the ER switch costs when switching from the neutral to the emotion task (which was the more difficult type of affective switching for our participants). Also, these three accuracy-based flexibility measures loaded on a common latent factor. However, given this latter finding results from an exploratory analysis and given that the ER-based switch cost measures have rather low reliabilities, we suggest considering these results as hypothesis-generating.

It could be questioned whether the mean number of errors after task reversal is an optimal indicator of individual differences in feedback-based flexibility. However, while it might be possible for neurophysiological methods like EEG to more directly tap into the process of implementing the new task set, there is no obvious behavioral indicator of that process. On the other hand, the fact that brain activation elicited during reversal errors involves fronto-parietal brain systems (Yaple & Yu, 2019) not unlike those activated during cue-instructed task switching (e.g., Kim et al., 2012) in our view suggests that the mean number of errors after task reversal is a reasonable proxy for feedback-based flexibility. This may receive further support from our observation that persons who spontaneously switch less often (in the cognitive flexibility paradigm) also in tendency need more trials before they adapt their behavior after a task reversal in the feedback-based task – both markers are here suggested as indicators of lower dispositional flexibility.

These results, at present, do not allow us to draw firm conclusions concerning whether or not feedback-based flexibility shares cognitive resources with purely cognitive or affective task set switching. Despite the procedural similarity in shifting between task sets, the cue-based and feedback-based paradigms also differ in several important aspects that may contribute to the so-called ‘task impurity problem’ (i.e., the danger that systematic variance stems from contextual variables relating to how the respective cognitive process is embedded into a specific task context, rather than to the cognitive function itself; Snyder et al., 2015). While instructed task switching can rely almost fully on external cues that signal the task to be performed, the feedback-based paradigm requires a substantial involvement of attentional and endogenous control processes involved in monitoring the task context and deriving an alternative task rule (Geurts et al., 2009; Logan, 2003). This also includes that participants have to learn from the received feedback (e.g., Stalnaker et al., 2015) and represent an estimate of the likelihood of an upcoming task reversal (e.g., Costa et al., 2015). Further research is needed to elucidate how these specific sub-processes might shape the association with cue-based task switching, its effectiveness and efficiency.

Our inconclusive results regarding the existence of a common latent flexibility factor may reflect greater heterogeneity in the different surface measures than initially expected, which may be consistent with the reported difficulty of fitting latent structures to experimentally assessed (executive) functions (see Karr et al., 2018 for a comprehensive discussion of latent models in executive functions). Karr and colleagues (2018) argue that sub-components of executive functions – like flexibility which is in the context of latent models of executive functions often termed ‘shifting’ – potentially contain a complex latent structure themselves, which may be in line with our findings here.

### Potential limitations of the present study

One possible reason for the absence of correlations among error rate (ER) switch cost measures is their relatively low reliability (which in turn can have detrimental effects on correlations among these variables). In addition (at least some of) the tasks may have been quite easy for our sample of student participants. However, the performance accuracy results are unlikely to represent a ceiling effect. Also, performance accuracy in the cognitive paradigm is comparable to that of a previous study with a student sample (n = 20; Armbruster et al., 2012) and worse than performance in a larger sample of randomly selected citizens of a broader age range (n = 95; 20–51 years; Armbruster-Genç et al., 2016). We thus consider it unlikely that the cue-based tasks were too easy to actually quantify individual differences in task switching performance.

On the other hand, participants made only between 1 and 3 reversal errors before switching in the feedback-based task (average: 1.72), which seems to be lower than in some previous studies with task reversals (e.g., Cools et al., 2002; Jocham et al., 2009; Waegeman et al., 2014). This may indicate either a relatively low task difficulty or the presence of strategic task performance, e.g., resulting from predictability of the task reversals. This may have reduced our chances to obtain correlative results involving feedback-based flexibility, which implies that we need larger sample sizes for future studies focusing on the potentially small correlations between feedback-based flexibility and cue-based task switching, or that task difficulty of the feedback-based flexibility paradigm should be increased (e.g., by introducing more conditions, increasing the length/variability of inter-switch intervals or the amount of probabilistic errors, or by reducing the predictability of switches). As already discussed, low reliability of the difference-based ER measures (switch costs) may also have influenced these results, as estimates for the mean reversal error can be considered moderate; future studies should therefore focus on RT or composite measures (Hughes et al., 2014).

Lastly, the lack of robust correlations among ER switch cost measures may also reflect a lack of power to detect weak correlations. The sample size of the present study was based on a power analysis which in turn was based on our theoretically derived minimum effect size of interest of *r* = .25 (one-tailed). However, recent publications have reported, in the domain of executive functions, lower correlations between tasks designed to measure the same latent variable (Gärtner & Strobel, 2019) or between tasks measuring different latent executive functions (Draheim et al., 2016). If one deems such weak associations important for understanding relationships among different facets of psychological flexibility, larger samples might be needed in future research.

### Implications for theories of psychological flexibility

The present study was motivated by a perceived need to clarify the inter-relationships among different facets of ‘psychological flexibility’ – which we propose as a conceptual frame for a number of constructs including various facets of cognitive, affective, behavioral, and feedback-based flexibility. Cognitive flexibility is impaired in psychiatric disorders like schizophrenia (e.g., Waltz, 2017), but has also been proposed as a protective factor, e.g., against the effects of stress and adversity on the development of affective disorders like depression (e.g., Haglund et al., 2007) or PTSD symptoms (Ben-Zion et al., 2018). The unclear correlative results for cue-instructed vs. feedback-based task switching indicate that it is at least problematic that both behaviors are often subsumed under the same label. This may hinder a refined understanding of the causes and consequences of individual differences in cognitive flexibility, and as a consequence also the specification of how the flexibility of thought and behavior relates to mental health (as discussed, e.g., for autism traits in Geurts et al., 2009). We thus propose that a more precise definition and discrimination of different facets of cognitive flexibility and related constructs is necessary. One step in this direction was described in an fMRI meta-analysis by Kim and colleagues (2012), who distinguished between perceptual switching (i.e., shifting attention between different perceptual features or dimensions of a stimulus), response switching (i.e., between arbitrary stimulus-response mappings), and context switching (equivalent to switches between task rules). The fractionation of processes proposed in this study is partly orthogonal to the present distinction of cue- vs. feedback-based flexibility, and combining these may offer an interesting theoretical framework.

A similar challenge arises for flexible behaviors in the face of affectively neutral vs. emotional stimuli. In the developmental literature, there has been a strong interest in the distinction between ‘cool’ (affectively neutral) and ‘hot’ (emotionally relevant) executive functions (e.g., Zelazo & Carlson, 2012). This has sometimes also been applied to propose separable brain systems for affective vs. affectively neutral neurocognitive processes in adults (e.g., Dolcos & McCarthy, 2006). Our results, however, do not provide strong support for this distinction, as task switching costs were quite robustly correlated between affective and affectively neutral domains. On the other hand, our study also yielded differential results for switching from neutral to emotional tasks and vice versa (i.e., asymmetric affective switch costs and a selective correlation with the number of reversal errors for the former switch type). This may be related to the difference in the attentional dominance of the emotional as compared to the neutral task (and resulting asymmetries in control demands; cf. Monsell et al., 2000; Reeck & Egner, 2015). Furthermore, theoretical considerations suggest that at least some of the processes underlying psychological flexibility might be context-specific (Braem & Egner, 2018) or related to superordinate goals of the individual (Doebel, 2019), which is not accounted for in the present work and thus could be addressed in future studies. A comprehensive model of psychological flexibility should address these methodological and theoretical aspects and aim at incorporating also other facets of dynamic, contextually adaptive behavior not addressed in the present study.

## Conclusion

To summarize, we here demonstrate a close association between instructed (i.e., cue-dependent) cognitive and affective task switching in terms of efficiency (response time switch costs), while feedback-based flexibility seems to be less closely related, mediated potentially by mechanisms that control the effectiveness of task switching (in terms of the accuracy of switching). These results are only partly compatible with a ‘simple’ model of a single underlying latent process that controls different aspects of psychological flexibility.

## Data Accessibility Statement

Raw data and analysis code will be made publicly available on https://osf.io/8f4cn/ by the authors upon publication.

## Additonal File

The additonal file for this article can be found as follows:

10.5334/joc.120.s1 Supplementary file 1. Appendix.
