# Supplementary material for: Cognitive, Affective, and Feedback-Based Flexibility – Disentangling Shared and Different Aspects of Three Facets of Psychological Flexibility
Source: J Cogn. 2020 Sep 9;3(1):21. doi: 10.5334/joc.120 (PMC7500224; doi:10.5334/joc.120)
Supplement: Supplementary file 1. — Appendix. [file joc-3-1-120-s1.pdf]

Cognitive, affective, and feedback-based flexibility – disentangling shared and different aspects of three facets of psychological flexibility

Dominik Kraft[1], Lena Rademacher[1,2], Cindy Eckart[1], Christian J. Fiebach[1, 3]

[1] Department of Psychology, Goethe University Frankfurt, Germany

[2] Department of Psychiatry and Psychotherapy, University of Lübeck, Lübeck, Germany

[3] Brain Imaging Center, Goethe University Frankfurt, Frankfurt am Main, Germany

Address for Correspondence:

Department of Psychology, Goethe University Frankfurt, Theodor-W.-Adorno-Platz 6, Frankfurt am Main 60323, Germany
Electronic address: kraft@psych.uni-frankfurt.de

Supplementary file 1: Appendix.

- Results from exploratory analysis without data exclusion (2 Tables)
- Results from partial correlation taking lifetime history of psychiatric history into account (2 Tables)

The following Tables S1 and S2 complement the pre-registered correlation analyses from the main paper, which implemented an exclusion of participants depending on their performance in the respective tasks (Tables 4 and 5). Here, we report in addition the correlation results for the full sample of N=100, i.e., without any exclusions (exploratory analysis).

Table S1. Pre-registered correlations for response time flexibility indices, spontaneous switch rate, and mean number of reversal errors for the full sample of N=100 (i.e., without exclusions; compare Table 1 in the main article).

	affective		***feedback***
	Switch cost RT (to emotion task)	Switch cost RT (to gender task)	reversal errors (*M*)
cognitive			
Switch cost RT	r_s =.35<br>p < .001<br>CI= [.16 - .51]	r = .48<br>p < .001<br>CI= [.31 - .62]	r_s = .12<br>p = .12
spontaneous switch rate	r_s = .004<br>p = .48	r_s = .08<br>p = .21	r_s = -.12<br>p = .11
affective			
Switch cost RT (to emotion task)			r_s = -.05<br>p = .32
Switch cost RT (to gender task)			r_s = .004<br>p = .48

RT= reaction time in millisecond; *M* =mean; CI = 95% confidence interval.

Table S2. Pre-registered correlations for accuracy-based flexibility indices and the mean number of reversal errors for the full sample of N=100 (i.e., without exclusions; compare Table 2 in the main article).

	affective		***feedback***
	Switch cost ER (to emotion task)	Switch cost ER (to gender task)	reversal errors (*M*)
cognitive			
Switch cost ER	r_s =.16<br>p = .06	r_s = .05<br>p = .30	r_s = .05<br>p = .31
affective			
Switch cost ER (to emotion task)			r_s = .28<br>p < .01<br>CI= [.09 - .45]
Switch cost ER (to gender task)			r_s = -.04<br>p = .33

M=mean; ER= error rate; CI= 95% confidence interval.

The following Tables S3 and S4 complement the pre-registered correlation analyses from the main paper (see Tables 4 and 5). Here, we calculated partial correlations taking into account that 10 participants reported a history of psychiatric disorder in their lifetime. We furthermore calculated whether participants with vs. without reported history of psychiatric disorder vs. participants who did not report any information differed with respect to the flexibility measures indicated in Table S3 and Table S4 but did not obtain any significant result (all $p > .23$).

Table S3. Partial Correlation for response time flexibility indices, spontaneous switch rate, and mean number of reversal errors taking into account the occurrence of psychiatric disorders in ten participants in their lifetime.

	affective		***feedback***
	Switch cost RT (to emotion task)	Switch cost RT (to gender task)	reversal errors (*M*)
cognitive			
Switch cost RT	r_s =.35<br>p < .001[a]	r = .51<br>p < .001[a]	r_s = .17<br>p = .05[b]
spontaneous switch rate	r_s = .03<br>p = .39 [d]	r_s = .06<br>p = .30 [d]	r_s = -.13<br>p = .13 [e]
affective			
Switch cost RT (to emotion task)			r_s = -.01<br>p = .47[c]
Switch cost RT (to gender task)			r_s = .03<br>p = .40[c]

Due to exclusion criteria, sample size varies across correlations; all correlations are one-sided; [a] N= 93; [b] N=94; [c] N=99; [d] N=82; [e] N=83; RT= reaction time in millisecond; *M* =mean.

Table S4. Partial Correlation for accuracy-based flexibility indices and mean number of reversal errors taking into account the occurrence of psychiatric disorders in ten participants in their lifetime.

	affective		***feedback***
	Switch cost ER (to emotion task)	Switch cost ER (to gender task)	reversal errors (*M*)
cognitive			
Switch cost ER	r_s =.17 p = .06[a]	r_s = .14 p = .09[a]	r_s = .03 p = .38[b]
affective			
Switch cost ER (to emotion task)			r_s = .28 p < .01 [c]
Switch cost ER (to gender task)			r_s = -.02 p = .41[c]

Due to exclusion criteria, sample size varies across correlations; all correlations are one-sided; [a] N= 93; [b] N=94; [c] N=99; *M*=mean; ER= error rate.
